# No evidence of hepatitis E virus (HEV) infection among pet cats and dogs, and low seroprevalence of hepatitis E virus among pet rabbits in Poland

**DOI:** 10.1007/s11259-023-10223-w

**Published:** 2023-09-23

**Authors:** Hanna Turlewicz-Podbielska, Jakub Jędrzej Ruszkowski, Jarosław Wojciechowski, Małgorzata Pomorska-Mól

**Affiliations:** 1 Department of Preclinical Sciences and Infectious Diseases, Faculty of Veterinary Medicine and Animals Sciences, Poznan University of Life Sciences, Wolynska 35, 60‑637, Poznan, Poland; 2 Department of Animal Anatomy, Faculty of Veterinary Medicine and Animals Sciences, Poznan University of Life Sciences, Wojska Polskiego 71C, 60‑625, Poznan, Poland; 3 VETPOL Sp. z o.o, Grabowa 3, Grudziadz, 86-300 Poland

**Keywords:** Cats, Dogs, Hepatitis E virus, HEV-3, Poland, Rabbits, Seroprevalence

## Abstract

The seroprevalence of *Paslahepevirus balayani* genotype 3 (hepatitis E virus genotype 3 – HEV-3; *Hepeviridae* family, genus *Paslahepevirus*) in pet cats, dogs and rabbits was evaluated. Samples from cats and dogs were collected from three veterinary practices from various parts of Poland: Poznan (wielkopolskie voivodeship), Przemysl (podkarpackie voivodeship) and Lublin (lubelskie voivodeship). Samples from rabbits were collected in Poznan. In total, serum samples from 90 cats, 82 dogs and 71 rabbits were selected and tested for specific anti-HEV-3 immunoglobulin (IgG) antibodies using a commercial ELISA test. Pathogen seroprevalence among rabbits was calculated at a 95% confidence interval (CI) for each gender, age (up to 12 months, 1–3 years, 4–7 years and over 8 years), symptoms group (healthy, gastrointestinal disorders, other disorders) and compared with a chi-squared test. No anti-HEV-3 IgG antibodies were detected in any of the samples from cats and dogs. Anti-HEV-3 IgG antibodies were detected in 2.82% of the serum samples from rabbits (2/71; 95% CI: 0.78–9.70). No significant correlations between seropositivity and gender, age, and symptoms (p > 0.05) were observed in rabbits. Our findings indicate that pet rabbits in Poland are exposed to HEV-3, develop humoral response due to infection and might constitute a source for HEV-3 transmission to humans.

## Introduction

*Paslahepevirus balayani* genotype 3 (hepatitis E virus genotype 3 – HEV-3) includes variants able to infect humans and several mammalian species. According to the 2021 release of the International Committee on the Taxonomy of Viruses, HEV belongs to the *Hepeviridae* family, *Orthohepevirinae* subfamily (divided into four genera: *Paslahepevirus*, *Avihepevirus*, *Rocahepevirus* and *Chirohepevirus*). Genus *Paslahepevirus* contains two species, *P. balayani* (formerly known as *Orthohepevirus* A) and *Paslahepevirus alci* (Purdy et al. 2022). Eight distinct genotypes thus far have been proposed within HEV with four major genotypes (1–4) implicated in human disease (Pirani et al. 2023). HEV-1 and HEV-2 infect only humans and cause acute hepatitis, predominantly in developing countries, where are spread orofaecally via contaminated water supplies (Dalton et al. 2014). HEV-3 and HEV-4 are well-recognized zoonotic strains that circulate among a broad spectrum of animal species, including pigs and wild boar, which are considered the main reservoirs (Li et al. 2022). In developed countries, HEV-3 and HEV-4 have been found in all stages of the human food chain and one established route of transmission from pigs to humans is via undercooked or uncooked pig meat products (Dalton et al. 2014). However, HEV-3 has also been found in soft fruits such as strawberries (Brassard et al. 2012). It can also be transmitted iatrogenic with blood transfusions or organ transplants (Dalton et al. 2014). Animal species other than pigs may also contribute to the spread of zoonotic HEV genotypes to humans. HEV-3 and HEV-4 RNA was detected in the liver (Wu et al. 2015; Li et al. 2017; Go et al. 2019) and milk (Huang et al. 2016; Demirci et al. 2019) samples collected from cows, goats and sheep. HEV-1, HEV-3 and HEV-4 were found in ruminants (Demirci et al. 2019). It poses a real risk of infection with zoonotic HEV genotypes after consuming infected unpasteurized milk or edible organs from these animals. Rabbits are the natural host of HEV-3 and are considered the main reservoir of HEV second to pigs (Wang et al. 2017b). Humans can acquire HEV-3 infection from rabbits via consumption of their undercooked meat or via contact with contaminated feces during viral shedding (Wang et al. 2017b). Human HEV infections are generally asymptomatic and self-limiting (Behrendt et al. 2014; Takakusagi et al. 2023). However, severe hepatitis in patients with concurrent hepatic disease and chronic hepatitis in immunocompromised patients may occur (Behrendt et al. 2014; Takakusagi et al. 2023). The mortality rate in humans is approximately 0.2–4.0% (Teshale et al. 2010; Takakusagi et al. 2023), however, rates of greater than 20% have been reported in pregnant women (Singh et al. 2003; Takakusagi et al. 2023). Zoonotic HEV-3 is circulating in Poland among human and pig populations (Grabarczyk et al. 2018; Bigoraj et al. 2021). RNA of HEV-3 was found in 2.4% (6/246) of samples of pig’s blood and liver for human consumption (Bigoraj et al. 2021). According to Grabarczyk et al. (2018), 43.5% (1340/3079) of human blood donors were positive for anti-HEV immunoglobulin G (IgG) antibodies. RNA of HEV-3 was identified in three individuals (Grabarczyk et al. 2018). The common prevalence rate of anti-HEV antibodies among cats and dogs in Europe and evidence of zoonotic HEV-3 circulation in the human and pig populations in Poland point to the need to evaluate the presence of anti-HEV-3 antibodies among pet animals in this country. Pets, including dogs, cats, and rabbits, are in close contact with humans and might be accidental hosts for HEV-3. Dogs and cats are carnivores and often eat raw porcine or cattle products intended for human consumption, so they can acquire HEV-3 infection. Rabbits are also likely to become infected by eating contaminated, unwashed fruit or vegetables. These animal species may indicate HEV-3 contamination of food products. Serological studies so far indicate that HEV (mainly HEV-3) circulates among cats, dogs, and rabbit populations worldwide (Di Bartolo et al. 2016; Dahnert et al. 2018; Lyoo et al. 2019; Li et al. 2020; Capozza et al. 2021; Caballero-Gomez et al. 2022; Cagigran et al. 2022). This study is aimed to investigate the evidence of infection with HEV-3 using a serological assay in domestic cats, dogs and rabbits in Poland.

## Materials and methods

### Samples

**Fig. 1:**
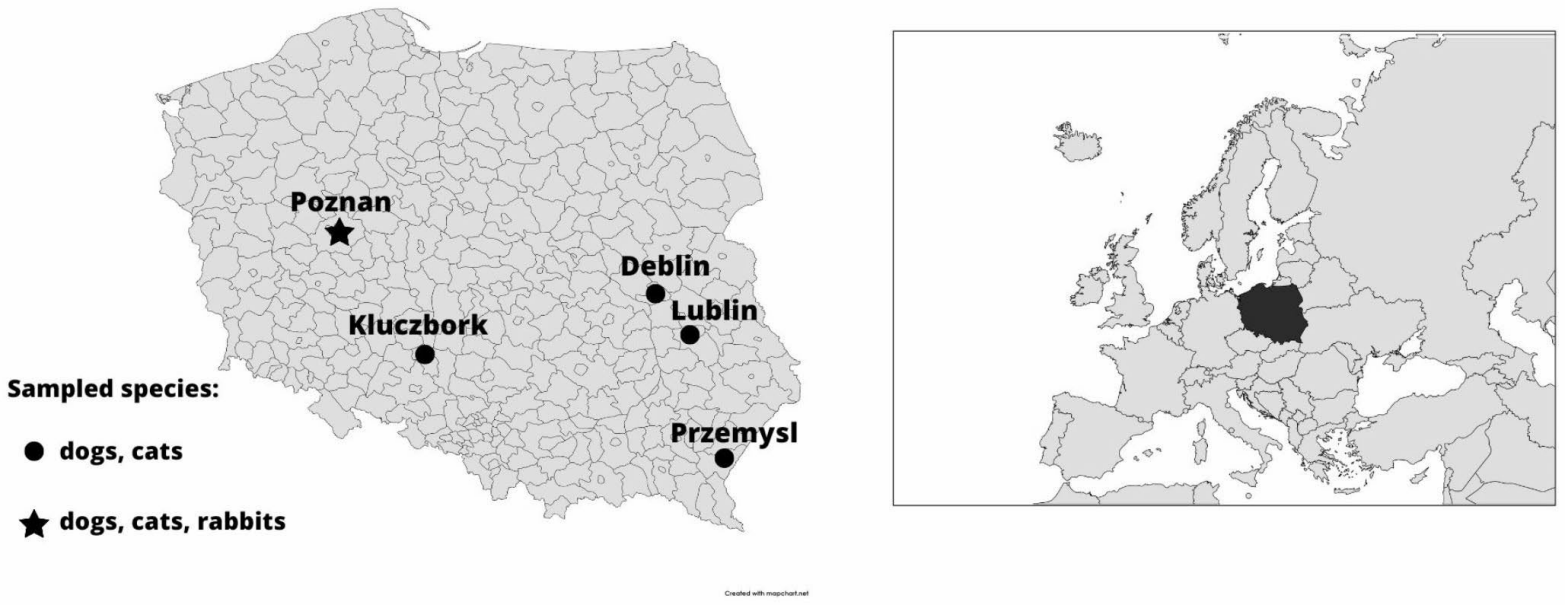
Map of sampling regions and species sampled. Poland is marked in black on the Europe map

A total of 243 serum samples collected between September 2020 and January 2022 in three veterinary practices located in various parts of Poland (Poznan 52°24′24″N 16°55′47″E (wielkopolskie voivodeship); Przemysl 49°47′05″N 22°46′02″E (podkarpackie voivodeship); Lublin 22°34′E 51°15′N (lubelskie voivodeship); (Fig. 1) were selected for this study. Samples from rabbits were collected in one practice (*n* = 71). Rabbit sera came from animals from Poznan and their surroundings. Sera were stored at − 70 °C until analyses. Serum samples from 90 cats, 82 dogs and 71 rabbits were used in the study. The following information has been available for each sample: species, gender, age at sampling, health status and location. Information about gender, age and health status was collected during anamnesis and clinical examination performed by veterinarians during the visits to veterinary clinics. The main reasons for the visits to the veterinary clinic other than gastrointestinal disorders were respiratory symptoms, chronic diseases and routine checks during vaccinations or before performing surgical procedures. The detailed information about the structure of a sampled population is presented in Table 1 (cats and dogs) and Table 2 (rabbits).

**Table 1:** The detailed information about the structure of the sampled cat and dog populations

Symptoms		Cats			Dogs
	Gastrointestinal disorders	13			19
	Others	58			36
	Healthy	19			27
Gender					
	Female	48			45
	Male	42			37
Age (years)					
	< 1	11			8
	1–3	23			14
	4–7	22			10
	8+	34			50
Location					
	Lublin	39			31
	Poznan	36			36
	Przemysl	15			15
Total		90			82

### Detection of anti-HEV-3 IgG antibodies

Serum samples were tested for the presence of anti-HEV-3 IgG antibodies using the ID Screen® Hepatitis E Indirect Multi-species ELISA, (IDvet, Grabels, France) according to the manufacturer’s instructions. This is a duplicate-well test, where even-numbered wells are coated with a recombinant antigen from the capsid of HEV-3, and odd-numbered wells are uncoated. Briefly, 10 µL of sera was diluted with 90 µL ELISA dilution buffer in the test plate and incubated for 45 min at room temperature (21°C +/- 5°C). The plate was washed three times, and the conjugate was added to each well. Then, the plate was incubated at the same temperature for 30 min and washed three times. Next, 100 µL of 3,3’,5,5’-tetramethylbenzidine (TMB) substrate solution was added to each well for 15 min, followed by 100 µL of stop solution. The optical density (OD) was measured at 450 nm in the Infinite® 200 PRO microplate reader (TECAN) immediately after stopping the reaction. The cut-off values (S/P%) that allowed the sample to be considered as positive, doubtful, or negative were calculated using the following formula: (optical density (OD) value of tested sample/OD value obtained for positive control) × 100%. The serum was considered positive when its cut-off value exceeded the borderline seropositivity of 70%. A doubtful result ranged from 60 to 70% and for negative sera, OD values were below 60%. The correct performance of the indirect ELISA was monitored using the results obtained for positive and negative control sera supplied with the assay.

### Statistical analysis

The analyses were performed using RStudio (version 4.1.2), except for prevalence (with 95% confidence intervals (CI)), which was available as an online program (https://epitools.ausvet.com.au/ciproportion). CI for prevalence was calculated with the Wilson score method. Pearson’s chi-square (*χ*2) tests were used to analyse the data in age groups, gender and symptoms in rabbits.

## Results

All of the serum samples from dogs and cats were negative for anti-HEV-3 IgG antibodies. The seroprevalence among rabbits was 2.82% (2/71; 95% CI: 0.78–9.70). No significant gender difference in seroprevalence between females and males and no significant differences regarding age group or symptoms exhibited by rabbits were found (Table 2).

**Table 2:** The number of seropositive individuals and prevalence of anti-HEV-3 antibodies in different groups of rabbits

		Positive/ examined	Prevalence (%)	95% CI^a^	p-value
Symptoms					
	Gastrointestinal disorders	0/2	0.00	0.00-65.76	0.74
	Other	0/25	0.00	0.00-13.32	
	Healthy	2/ 44	4.55	1.26–15.13	
Gender					
	Female	2/39	5.13	1.42–16.89	0.43
	Male	0/29	0.00	0.00-11.7	
	No data available	0/3			
Age group					
	< 1	1/16	6.25	1.11–28.33	0.88
	1–3	0/21	0.00	0.00-15.46	
	4–7	1/22	4.55	0.81–21.80	
	8+	0/7	0.00	0.00-35.43	
	No data available	0/5			
Total		2/71	2.82	0.78–9.70	

^a^95% CI: lower and upper values for the 95% confidence interval

## Discussion

Cats, dogs and rabbits are animal species commonly kept as pets worldwide. Moreover, these species are often kept at home together and usually have close contact in the same household. Several studies suggest that pets are frequently exposed to HEV-3 or HEV-4 and thus are potentially infectious for humans in close contact (Lyoo et al. 2019; Mrzljak et al. 2021). The sporadic acute hepatitis E of a 47-year-old man whose pet cat was positive for the antibody to hepatitis E virus was reported in 2003 and it was the first suggestion that HEV has a zoonotic potential (Kuno et al. 2003). Veterinarians, who have close contact with cats, dogs and rabbits, are supposed to have higher rates of anti-HEV antibody positivity compared to the general population (Lyoo et al. 2019; Mrzljak et al. 2021). Moreover, having a pet at home was identified as a risk factor for HEV seropositivity in several studies (Hriskova et al. 2021).

HEV was identified for the first time in a pet house rabbit (an adult 7 years old female) in 2015. Rabbit liver tested positive by real-time PCR (Caruso et al. 2015). Rabbits may have become infected with HEV-3 in several ways: consumption of contaminated vegetables and fruits or contaminated water. The hay provided to pet owners by an organic farm could also be considered a primary source of infection (Caruso et al. 2015). Laboratory rabbits infected with HEV-3 or HEV-4 could present similar signs of acute and chronic hepatitis E in humans (Wang et al. 2018). After experimental infection of specific-pathogen-free rabbits with HEV-3, Wang et al. (2017b) observed viremia and fecal shedding. Fecal shedding lasts up to 40 weeks. The infection led to kidney and liver injury (Wang et al. 2017b). It cannot be excluded that humans may become infected with HEV-3 or HEV-4 when cleaning litter boxes or through contact with infected feces from rabbits. There are several cases of humans infected with a strain of HEV that is genetically similar to those found in rabbits (Kaiser et al. 2018; Sooryanarain and Meng 2019).

The available data on HEV seroprevalence among pet rabbits is scarce. The seroprevalence among farm and pet rabbits in Italy reached 3.40% (7/206) and 6.56% (8/122) respectively (Di Bartolo et al. 2016). However, in a recent study conducted in Italy, 328 hares and 59 farmed rabbits were screened for anti-HEV antibodies and none of them were positive (De Sabato et al. 2023). In Poland, antibodies against HEV were detected in 6% (29/482) of breeding rabbits (Bigoraj et al. 2020). The seroprevalence in our study reached 2.82% (95% CI: 0.78–9.70). Presumably, the older age of animals could increase the likelihood of exposure to HEV-3 and seroconversion; however, statistical analysis of our results showed that the correlation between the age of rabbits and the presence of anti-HEV-3 IgG antibodies was not significant (Table 2). Animals examined in our study were assigned to five age groups. Unfortunately, the age of 5 rabbits was not known. Two seropositive rabbits were found in two age groups: one in the age group below 12 months and the second in the age group of 4–7 years. Seventy-one rabbit sera were tested, which is relatively a small size sample. These samples were also collected from one region and our results may not fully represent the real prevalence in Poland. However, our results indicate that HEV-3 circulates among pet rabbits in Poland and these animals may play a potential role in the epidemiology of HEV-3 infection. To the author’s best knowledge, this is the first study concerning HEV seroprevalence among pet rabbits in Poland.

According to serological studies conducted in Europe, the prevalence of anti-HEV antibodies in stray and pet Spanish cats was 2.8% (4/144) (Caballero-Gomez et al. 2022). The seroprevalence in pet cats in Europe was 32.3% (21/65) in Germany (Dahnert et al. 2018), 14.9% (7/47) in the Netherlands (Li et al. 2020), 3.1% (10/324) in Italy (Capozza et al. 2021), and 5.4% (5/91) in Türkiye (Cagigran et al. 2022). The seroprevalence in stray and pet Spanish dogs was 9.9% (15/152) (Caballero-Gomez et al. 2022). In pet dogs, the seroprevalence reached 18.52% (30/162) in the Netherlands (Li et al. 2020) and 56.6% (47 of 83) in Germany (Dahnert et al. 2018). No anti-HEV-3 antibodies were detected in either cats or dogs in our study. Our results suggest that HEV-3 infections in these populations are rare, although our results may not reflect real prevalence throughout the country.

This study has some limitations that should be kept in mind. We have searched for evidence of HEV-3 infection via one serological method (indirect ELISA) and did not use species-specific HEV-3 immunoassays. Cross-reactivity to the related *hepevirus* cannot be ruled out despite the high specificity of the ELISA used in the present study. However, serological assays are preferable to a broad screening of potential animal reservoirs of HEV. HEV-specific antibodies are detectable over a much longer period of time than viral RNA, hence the detection window is wide (Panda et al. 2007). Further studies testing serum, including western blot, and molecular analyses of the sera and fecal samples should be carried out to increase the sensitivity of HEV-3 detection in these species. Additionally, a full understanding of the epidemiology of HEV-3 infection in humans and other animals requires a full-length HEV-3 genome isolated from pets.

We have not found evidence of exposure to HEV-3 in dogs and cats. The results of our study suggest that rabbits can become infected and develop a humoral response in response to infection with HEV-3. These results are beneficial to researchers, health workers, and veterinary practitioners. Considering the routes of infection in rabbits, good hygiene practices, washing fruits and vegetables, and giving hay and water from a verified source are the primary ways to prevent HEV-3 infection. The knowledge of good hygiene practices for the pet owner is crucial in the prevention of zoonotic hepatitis E. Even if the true potential of HEV-3 transmission from rabbits to humans is not known and further issues remain to be clarified, our data proved that pet rabbits should be considered in the epidemiological scenario of HEV-3 infection and this issue requires more in-depth research.

## Data Availability

The data that support the findings of this study are not openly available but are available from the corresponding author upon reasonable request.
